# Finding Provably Optimal Markov Chains

**DOI:** 10.1007/978-3-030-72016-2_10

**Published:** 2021-03-01

**Authors:** Jip Spel, Sebastian Junges, Joost-Pieter Katoen

**Affiliations:** 8 Eindhoven University of Technology, Eindhoven, The Netherlands; 9 Aalborg University, Aalborg East, Denmark; 10 RWTH Aachen University, Aachen, Germany; 11 University of California, Berkeley, California USA

## Abstract

Parametric Markov chains (pMCs) are Markov chains with symbolic (aka: parametric) transition probabilities. They are a convenient operational model to treat robustness against uncertainties. A typical objective is to find the parameter values that maximize the reachability of some target states. In this paper, we consider automatically proving robustness, that is, an $\varepsilon$-close upper bound on the maximal reachability probability. The result of our procedure actually provides an almost-optimal parameter valuation along with this upper bound.

We propose to tackle these ETR-hard problems by a tight combination of two significantly different techniques: monotonicity checking and parameter lifting. The former builds a partial order on states to check whether a pMC is (local or global) monotonic in a certain parameter, whereas parameter lifting is an abstraction technique based on the iterative evaluation of pMCs without parameter dependencies. We explain our novel algorithmic approach and experimentally show that we significantly improve the time to determine almost-optimal synthesis.

## 1 Introduction

*Background and problem setting.* Probabilistic model checking [3, 20] is a well-established field and has various applications but assumes probabilities to be fixed constants. To deal with uncertainties, symbolic parameters are used. Parametric Markov chains (pMCs, for short) define a family of Markov chains with uncountably many family members, called instantiations, by having symbolic (aka: parametric) transition probabilities [10, 22]. We are interested in determining optimal parameter settings: which instantiation meets a given objective the best? The typical objective is to maximize the reachability probability of a set of target states. This question is inspired by practical applications such as: *what are the optimal parameter settings in randomised controllers to minimise power consumption?*, and *what is the optimal bias of coins in a randomised distributed algorithm to maximise the chance of achieving mutual exclusion?* For most applications, it suffices to achieve parameters that attain a given quality of service that is

* Supported by DFG RTG 2236 "UnRAVeL" and ERC AdG 787914 FRAPPANT.
** Supported by the NSF grants 1545126 (VeHICaL) and 1646208, by the DARPA Assured Autonomy program, by Berkeley Deep Drive, and by Toyota under the iCyPhy center.

$\varepsilon$-close to the *unknown* optimal solution. More precisely, this paper concentrates on automatically proving $\varepsilon$*-robustness*, i.e., determine an upper bound which is $\varepsilon$-close to the maximal reachability probability. The by-product of our procedure actually provides an *almost-optimal* parameter valuation too.

*Existing parameter synthesis techniques.* Efficient techniques have been developed in recent years for the feasibility problem: given a parametric Markov chain, and a reachability objective, find an instantiation that reaches the target with at least a given probability. To solve this problem, it suffices to "guess" a correct family member, i.e., a correct parameter instantiation. Verifying the "guessed" instantiation against the reachability objective is readily done using off-the-shelf Markov chain model-checking algorithms. Most recent progress is based on advanced techniques that make informed guesses: This ranges from using sampling techniques [14], guided sampling such as particle swarm optimisation [7], by greedy search [24], or by solving different variants of a convex optimisation problem around a sample [8, 9]. Sampling has been accelerated by reusing previous model checking results [25], or by just in time compilation of the parameter function [12]. These methods are inherently inadequate for finding *optimal* parameter settings. To the best of our knowledge, optimal parameter synthesis has received scant attention so far. A notable exception is the analysis (e.g., using SMT techniques) of rational functions, typically obtained by some form of state elimination [10,12,15], that symbolically represent reachability probabilities in terms of the parameters. These functions are exponential in the number of parameters [16] and become infeasible for more than two parameters. Parameter lifting [5, 6, 25] remedies this by using an abstraction technique, but due to an exponential blow-up of region splitting, is limited to a handful of parameters. *The challenge is to solve optimal parameter synthesis problems with more parameters.*

*Approach.* We propose to tackle the optimal synthesis problem by a deep integration of two seemingly unrelated techniques: *monotonicity checking* [27] and *parameter lifting* [25]. The former builds a partial order on the state space to check whether a pMC is (local or global) monotonic in a certain parameter, while the latter is an abstraction technique that "lifts" the parameter dependencies, obtaining interval MCs [17,21], and solves them in an iterative manner. To construct an efficient combination, we extend both methods such that they profit from each other. This is done by combining them with a tailored *divide-and-conquer* component, see Fig. 1. To prove bounds on the induced reachability probability, parameter lifting has been the undisputed state-of-the-art, despite the increased attention that parameter synthesis has received over recent years. This paper improves parameter lifting with more advanced reasoning capabilities that involve properties of the derivative, rather than the actual probabilities. These reasoning methods enable reducing the exponent of the inherently exponential-time procedure. This conceptual advantage is joined with various engineering efforts. Parameter lifting is accelerated by using side products of monotonicity analysis such as local monotonicity and shrinked parameter regions. Furthermore, bounds obtained by parameter lifting are used to obtain a cheap rule accelerating the

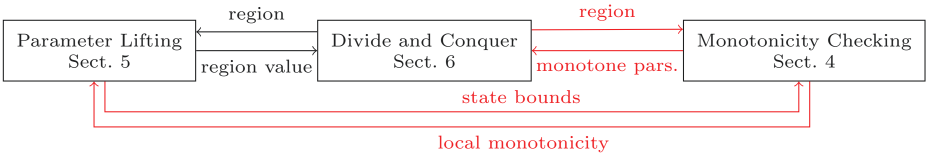

**Fig. 1.** The symbiosis of parameter lifting and monotonicity checking. Red are new interactions, compared to earlier work. Details are given in Sect. 3.

monotonicity checker. The interplay between the two advanced techniques is tricky and requires a careful treatment.

Note that we are not the first to exploit monotonicity in the context of pMCs. Hutschenreiter *et al.* [16] showed that the complexity of model checking (a monotone fragment of) PCTL on monotonic pMC is lower than on general pMCs. Pathak *et al.* [24] provided an efficient greedy approach to repair monotonic pMCs. Recently, Gouberman *et al.* [13] used monotonicity for hitting probabilities in perturbed continuous-time MCs.

*Experimental results.* We realised the integrated approached on top of the Storm [11] model checker. Experiments on several benchmarks show that optimal synthesis is possible: (1) on benchmarks with up to about a few hundred parameters, (2) on benchmarks that cannot be handled without monotonicity, (3) while accelerating pure parameter lifting by up to two orders of magnitude. Our approach induces a bit of overhead on small instances for some benchmarks, and starts to pay off when increasing the number of parameters.

*Main contribution.* In summary, the main contribution of this paper is a tight integration of parameter lifting and monotonicity checking. Experiments indicate that this novel combination substantially improves upon the state-of-the-art in *optimal* parameter synthesis.

*Organisation of the paper.* Section 2 provides the necessary technical background and formalises the problem. Section 3 explains the approach—in particular the meaning of the arrows in Fig. 1. Section 4 discusses how to state bounds can be exploited in the monotonicity checker. Section 5 details how to exploit local monotonicity in parameter lifting. Section 6 then considers the tight interplay via the divide-and-conquer method. Section 7 reports on the experimental results of our prototypical implementation in Storm while Section 8 concludes the paper.

## 2 Problem Statement

A *probability distribution* over a finite or countably infinite set $X$ is a function $\mu \colon X \rightarrow [0, 1] \subseteq \mathbb{R}$ with $\sum_{x \in X} \mu(x) = 1$. The set of all distributions on $X$ is denoted by $Distr(X)$. Let $\vec{a} \in \mathbb{R}^n$ denote $(a_1, \ldots, a_n)$. The set of multivariate polynomials over ordered variables $\vec{x} = (x_1, \ldots, x_n)$ is denoted $\mathbb{Q}[\vec{x}]$. For a polynomial $f$ and variable $x$, we write $x \in f$ if the variable occurs in the polynomial $f$. An *instantiation* for a finite set $V$ of real-valued variables is a function $u\colon V \to \mathbb{R}$. We often denote $u$ as a vector $\vec{u} \in \mathbb{R}^n$ with $u_i := u(x_i)$ for $x_i \in V$. A polynomial $f$ can be interpreted as a function $f\colon \mathbb{R}^n \to \mathbb{R}$, where $f(\vec{u})$ is obtained by substitution, i.e., $f[\vec{x} \leftarrow \vec{u}]$, where each occurrence of $x_i$ in $f$ is replaced by $u(x_i)$.

**Definition 1 (pMC).** *A* parametric Markov Chain (pMC) *is a tuple* $\mathcal{M} = (S, s_I, T, V, \mathcal{P})$ *with a finite set* $S$ *of* states*, an* initial state $s_I \in S$*, a finite set* $T \subseteq S$ *of* target states*, a finite set* $V$ *of real-valued variables* (parameters) *and a* transition function $\mathcal{P}\colon S \times S \to \mathbb{Q}[V]$.

A pMC $\mathcal{M}$ is a *(discrete-time) Markov chain* (MC) if the transition function yields *well-defined* probability distributions, i.e., $\mathcal{P}(s, \cdot) \in \mathit{Distr}(S)$ for each $s \in S$. Applying an *instantiation* $\vec{u}$ to a pMC $\mathcal{M}$ yields $\mathcal{M}[\vec{u}]$ by replacing each $f \in \mathbb{Q}[V]$ in $\mathcal{M}$ by $f(\vec{u})$. An instantiation $\vec{u}$ is *well-defined* (for $\mathcal{M}$) if $\mathcal{M}[\vec{u}]$ is an MC. A well-defined instantiation $\vec{u}$ is *graph-preserving* (for $\mathcal{M}$) if the topology is preserved, i.e., $\mathcal{P}(s, s') \neq 0$ implies $\mathcal{P}(s, s')(\vec{u}) \neq 0$ for all states $s$ and $s'$. A set of instantiations is called a *region*. A region $R$ is well-defined (graph-preserving) if $\vec{u}$ is well-defined (graph-preserving) for all $\vec{u} \in R$. In this paper, we consider only graph-preserving regions.

For a parameter-free MC $\mathcal{M}$, $\Pr^s_{\mathcal{M}}(\lozenge T) \in [0, 1] \subseteq \mathbb{R}$ denotes the probability that from state $s$ the target $T$ is eventually reached. For a formal definition, we refer to, e.g., [4, Ch. 10]. For pMC $\mathcal{M}$, $\Pr^s_{\mathcal{M}}(\lozenge T)$ is not a constant, but rather a function $\mathsf{Pr}^{s \to T}_{\mathcal{M}}\colon V \to [0, 1]$, with $\mathsf{Pr}^{s \to T}_{\mathcal{M}}(\vec{u}) = \Pr^s_{\mathcal{M}[\vec{u}]}(\lozenge T)$. The closed-form of $\mathsf{Pr}^{s \to T}$ on a graph-preserving region is a rational function over $V$, i.e., a fraction of two polynomials over $V$. On a graph-preserving region, the function $\mathsf{Pr}^{s \to T}$ is continuously differentiable [25]. We call $\mathsf{Pr}^{s \to T}_{\mathcal{M}}$ the *solution function*, and for conciseness, we often omit the subscript $\mathcal{M}$. Graph-preserving instantiations $\vec{u}, \vec{u}'$ preserve zero-one probabilities, i.e., $\mathsf{Pr}^{s \to T}(\vec{u}) = 0$ implies $\mathsf{Pr}^{s \to T}(\vec{u}') = 0$, and analogous for $=1$. We simply write $\mathsf{Pr}^{s \to T} = 0$ (or $=1$). Let ☺ (☹) denote all states $s \in S$ with $\mathsf{Pr}^{s \to T} = 1$ ($\mathsf{Pr}^{s \to T} = 0$). By a standard preprocessing [4], we may safely assume a single ☺ and ☹ state.

*Problem statement.* This paper is concerned with the following questions for a given pMC $\mathcal{M}$ with target states $T$, and region $R$:

*Optimal synthesis.* Find the instantiation $\vec{u}^*$ such that

$$\vec{u}^* = \arg\max_{\vec{u} \in R} \Pr_{\mathcal{M}[\vec{u}]}(\lozenge T)$$

*$\epsilon$-Robustness.* Given tolerance $\varepsilon \geq 0$, find an instantiation $\vec{u}^*$ such that

$$\max_{\vec{u} \in R} \Pr_{\mathcal{M}[\vec{u}]}(\lozenge T) - \varepsilon \leq \Pr_{\mathcal{M}[\vec{u}^*]}(\lozenge T) \leq \max_{\vec{u} \in R} \Pr_{\mathcal{M}[\vec{u}]}(\lozenge T) .$$

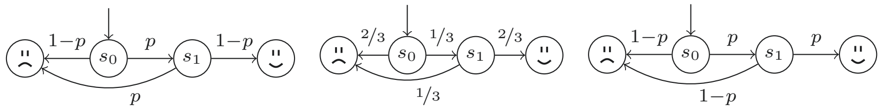

(a) $\mathcal{M}_1$ (b) $\mathcal{M}_1[\vec{u}]$, $\vec{u} = \{p \mapsto 1/3\}$ (c) $\mathcal{M}_2$

**Fig. 2.** Toy examples for pMCs.

The optimal synthesis problem is ETR-hard [28], i.e., as hard as finding a root of a multivariate polynomial. It is thus NP-hard and in PSPACE. The same applies to $\varepsilon$-robustness. The value of $\lambda$ can be viewed as the optimal reachability probability of $T$ — up to the robustness tolerance $\varepsilon$ — over all possible parameter values while $\vec{u}^*$ is the instantiation that maximises the probability to reach $T$.

Like [28], we assume pMCs to be *simple*, i.e., $\mathcal{P}(s, s') \in \{x, 1{-}x \mid x \in V\} \cup \mathbb{Q}$ for all $s, s' \in S$ and $\sum_{s'} \mathcal{P}(s, s') = 1$. Theoretically, the above problem for simple pMCs is as hard as for general pMCs, and practically, most pMCs are simple. For simple pMCs, the graph-preserving instantiations are in $(0, 1)^{|V|}$. Regions are assumed to be *well-defined*, *rectangular* and closed, i.e., a region is a Cartesian product of closed intervals, $R = \bigtimes_{x \in V} [\ell_x, u_x]$. Let $R(x)$ denote the interval $[\ell_x, u_x]$ and $\mathsf{occur}(s)$ the set of variables $\{x \in V \mid \exists s' \in S.\ x \in \mathcal{P}(s, s')\}$. For simple pMCs, this set has cardinality at most one. A state $s$ is called *parametric*, if $\mathsf{occur}(s) \neq \emptyset$; we write $\mathsf{occur}(s) = x$ if $\{x\} = \mathsf{occur}(s)$.

*Example 1.* Fig. 2(a) depicts a pMC. A region $R$ is given by $p \in [1/4, 1/2]$. An instantiation $\vec{u} = \{p \mapsto 1/3\} \in R$ yields the pMC in Fig. 2(b). The solution function is $\mathsf{Pr}^{s_0 \to T}_{\mathcal{M}_1} = p \cdot (1 - p)$. Indeed $\mathsf{Pr}^{s_0 \to T}_{\mathcal{M}_1}(\vec{u}) = 2/9 = \mathrm{Pr}_{\mathcal{M}_1[\vec{u}]}(\lozenge T)$.

## 3 Main Ingredients in a Nutshell

To solve the problem statement, we consider an iterative method which analyzes regions, and, if necessary, splits these regions. In particular, we combine two approaches — parameter lifting and monotonicity checking — as shown in Fig. 1.

### 3.1 The Monotonicity Checker

We consider *local* and *global* monotonicity. We start with defining the latter.

**Definition 2 (Global monotonicity).** *A continuously differentiable function $f$ on region $R$ is* monotonic increasing *in variable $x$, denoted $f{\uparrow}^R_x$, if $\frac{\partial}{\partial x} f(\vec{u}) \geq 0$ for all $\vec{u} \in R^3$. The pMC $\mathcal{M} = (S, s_I, T, V, \mathcal{P})$ is* monotonic increasing *in parameter $x \in V$ on graph-preserving region $R$, written $\mathcal{M}{\uparrow}^R_x$, if $\mathsf{Pr}^{s_I \to T}{\uparrow}^R_x$.*

[3] To be precise, on the interior of the closed set $R$.

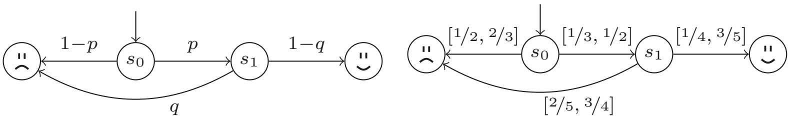

(a) $\mathcal{M}_3$ (b) $\mathcal{M}_3$, $R$ s.t. $p \in [1/3, 1/2]$, $q \in [2/5, 3/4]$

**Fig. 3.** Simple pMC that indeed is an iMC.

Monotonic *decreasing*, written $\mathcal{M}{\downarrow}_x^R$, is defined analogously. Let $\mathsf{succ}(s) = \{s' \in S \mid \mathcal{P}(s,s') \neq 0\}$ be the set of direct successors of $s$. Given the recursive equation $\mathsf{Pr}^{s \to T} = \sum_{s' \in \mathsf{succ}(s)} \mathcal{P}(s,s') \cdot \mathsf{Pr}^{s' \to T}$ for state $s \neq$ ☺, ☹, we have

$$\mathcal{M}{\uparrow}_x^R \quad \text{iff} \quad \frac{\partial}{\partial x}\left(\sum_{s' \in \mathsf{succ}(s)} \mathcal{P}(s,s') \cdot \mathsf{Pr}^{s' \to T}\right)(\vec{u}) \geq 0 \ ,$$

for all $\vec{u} \in R$. Rather than checking global monotonicity, the monotonicity checker determines a subset of the *locally* monotone state-parameter pairs. Such pairs intuitively capture monotonicity of a parameter only locally at a state $s$.

**Definition 3 (Local monotonicity).** *Function* $\mathsf{Pr}^{s \to T}$ *is* locally monotonic increasing *in parameter $x$ (at state $s$) on region $R$, written* $\mathsf{Pr}^{s \to T}{\uparrow}_x^{\ell,R}$, *if*

$$\forall \vec{u} \in R. \qquad \left(\sum_{s' \in succ(s)} \left(\frac{\partial}{\partial x}\mathcal{P}(s,s')\right) \cdot \mathsf{Pr}^{s' \to T}\right)(\vec{u}) \geq 0.$$

Thus, while global monotonicity considers the derivative of the entire solution function, local monotonicity (in $s$) only considers the derivative of the first transition (emanating from $s$). Local monotonicity of parameter $x$ in every state implies global monotonicity of $x$, as shown in [27]. As checking global monotonicity is co-ETR hard [27], a practical approach is to check *sufficient conditions* for monotonicity. These conditions are based on constructing a pre-order on the states of the pMC; this is explained in detail in Section 4.

*Example 2.* For $R = \{\vec{u}(p) \in [1/10, 9/10]\}$, pMC $\mathcal{M}_1$ in Fig. 2(a) is locally monotonic increasing in $p$ at $s_0$ and locally monotonic decreasing in $p$ at $s_1$. From this, we cannot conclude anything about global monotonicity of $p$ on $R$. Indeed, the pMC is not globally monotonic on $R$. $\mathcal{M}_1$ is globally monotonic on $R' = \{\vec{u}(p) \in [1/10, 1/2]\}$, but this cannot be concluded from the statement above. Contrarily, the pMC $\mathcal{M}_2$ in Fig. 2(c) is locally monotonic increasing in $p$ at both $s_0$ and $s_1$, and is therefore globally monotonic increasing in $p$.

### 3.2 The Parameter Lifter

The key idea of parameter lifting [25] is to drop all parameter dependencies—parameters that occur at multiple states in a pMC—by introducing fresh parameters. The outcome is an *interval* Markov chain [17,21], which can be considered a special case of pMCs in which no parameter occurs at multiple states.

**Definition 4 (Interval MC).** *A pMC is a (simple)* interval MC (iMC)*, if* $\mathsf{occur}(s) \cap \mathsf{occur}(s') = \emptyset$ *for all states* $s \neq s'$*.*

All iMCs in this paper are simple. We typically label transitions emanating from state $s$ in an iMC with $x = \mathsf{occur}(s)$ by $R(x) = [\ell_x, u_x]$.

*Example 3.* The pMC in Fig. 3(a) is an iMC. For a fixed $R$, the typical notation is given in Fig. 3(b). For the pMC $\mathcal{M}_1$ in Fig. 2(a), the parameter $p$ occurs at states $s_0$ and $s_1$, so that this pMC is not an iMC.

**Definition 5 (Relaxation).** *The* relaxation *of simple pMC* $\mathcal{M}=(S,s_I,T,V,\mathcal{P})$ *is the iMC* $\mathsf{relax}(\mathcal{M}) = (S, s_I, T, V', \mathcal{P}')$ *with* $V' = \{x_s \mid s \in S, \mathsf{occur}(s) \neq \emptyset\}$, $\mathcal{P}'(s, s') = \mathcal{P}(s, s')[\mathsf{occur}(s) \leftarrow x_s]$.

*For state* $s$ *with* $\mathsf{occur}(s) = x$*, let* $\mathsf{relax}(R)(x_s) = R(\mathsf{occur}(s))$*. Likewise, an instantiation in* $\vec{u} \in R$ *is mapped to* $\mathsf{relax}(\vec{u})$ *by* $\mathsf{relax}(\vec{u})(x_s) = \vec{u}(\mathsf{occur}(s))$*.*

Extremal reachability probabilities on iMCs are reached at the extremal values of a region. Formally [25], for each state $s$ and region $R$ in pMC $\mathcal{M}$:

$$\max_{\vec{u} \in R} \mathsf{Pr}^{s \to T}_{\mathcal{M}}(\vec{u}) \;\leq\; \max_{\vec{u} \in \mathsf{relax}(R)} \mathsf{Pr}^{s \to T}_{\mathsf{relax}(\mathcal{M})}(\vec{u}). \tag{1}$$

This result is a direct consequence of local monotonicity at all states implying global monotonicity. The extremal values for the reachability probabilities in the obtained iMCs are obtained by interpreting the iMCs as MDPs and applying off-the-shelf MDP model checking. We denote the right-hand side of (1) as upper bound on $R$, denoted $U_R(s)$. Analogously we define a lower bound $L_R(s)$.

*Example 4.* The pMC $\mathcal{M}_3$ in Fig. 3(a) is the relaxation of the pMC $\mathcal{M}_1$ in Fig. 2(a). Indeed, for $R = \{\vec{u}(p) \in [1/4, 3/4]\}$:

$$\max_{\vec{u} \in R} \mathsf{Pr}^{s_0 \to T}_{\mathcal{M}_1}(\vec{u}) = 1/4 \;\leq\; 9/16 = \max_{\vec{u} \in \mathsf{relax}(R)} \mathsf{Pr}^{s_0 \to T}_{\mathcal{M}_3}(\vec{u}).$$

### 3.3 Divide and Conquer

Figure 4 shows how the extremal value for region $R_\iota$, pMC $\mathcal{M}$, reachability property $\varphi$ and precision $\epsilon$ can be computed *using only parameter lifting* [25]: This paper extends this iterative approach to include monotonicity checking. The main idea is to analyze regions and split them if the result is inconclusive. The approach uses a queue of regions that need to be checked and the current extremal value `CurMax` found so far. In particular, we maintain a lower bound on `CurMax` and know a (potentially trivial) upper bound: $(\texttt{CurMax}+\varepsilon) \geq \max_{\hat{R} \in Q} U_{\hat{R}}(s_I)$. We iteratively check regions and improve both bounds until a satisfactory solution is found. Initially, the queue only contains $R_\iota$. For a selected $R$ from the queue we compute an upper bound $U_R$ with parameter lifting. If $U_R$ at the initial state

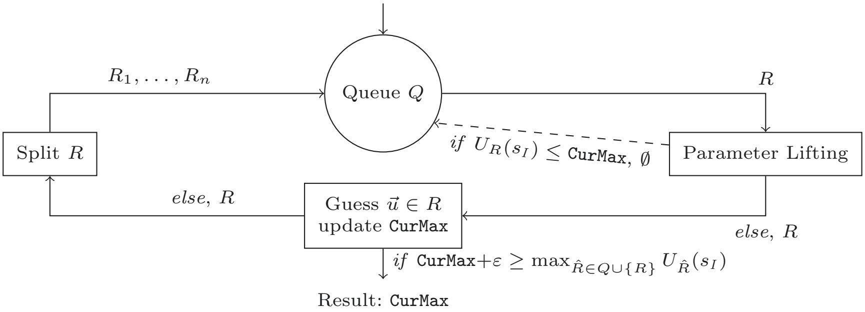

**Fig. 4.** Divide and conquer with pure parameter lifting is below the current optimum, we can safely discard $R$. Otherwise, we want attempt to improve `CurMax` by guessing $u \in R$ and computing $\mathsf{Pr}_{\mathcal{M}}^{s \to T}(\vec{u})$ using model checking[4]. If $\mathsf{Pr}_{\mathcal{M}}^{s \to T}(\vec{u})$ exceeds `CurMax`, we update `CurMax`. Now, we check whether we can terminate:

In particular, let the maximum so far be bounded by $\max_{\hat{R} \in Q \cup \{R\}} U_{\hat{R}}(s_I)$. If the upper bound is below `CurMax`$+\varepsilon$, we are done, and return `CurMax` together with the $u$ associated with `CurMax`. Otherwise, we continue and split $R$ into smaller regions. By default, parameter lifting splits $R$ along all dimensions. This algorithm converges in the limit [25].

*Example 5.* Reconsider Ex. 4, and assume we want to show $\max_{\vec{u} \in R} \mathsf{Pr}_{\mathcal{M}_1}^{s_0 \to T}(\vec{u}) \leq {}^1\!/_4$, with $\varepsilon = {}^1\!/_8$. We sample in (the middle of) $R$ and obtain `CurMax` $= {}^1\!/_4$, while the upper bound $U_R(s_I)$ from Ex. 4 is ${}^9\!/_{16}$. We split $R$ into two regions $R_1 = \{\vec{u}(p) \in [{}^1\!/_4, {}^1\!/_2]\}$ and $R_2 = \{\vec{u}(p) \in [{}^1\!/_2, {}^3\!/_4]\}$. Parameter lifting reveals that for both regions the bound is ${}^3\!/_8$. Thus, ${}^1\!/_4$ is an epsilon-close instance.

The remainder of this paper integrates monotonicity checking in this loop.

This paper addresses **three challenges**: (Sect. 4): Using state bounds in the monotonicity checker. (Sect. 5): Using local monotonicity in parameter lifting. (Sect. 6) Integrating monotonicity in the divide and conquer loop.

## 4 A New Rule for Sufficient Monotonicity

As discussed in Section 3.1, we aim to analyse whether for a given region $R$, parameter $x$ is locally monotonic at state $s$. The key ingredient is a pre-order on the states of the pMC at hand that is used for checking sufficient conditions for being local monotonic. We define the pre-order and recap the "cheap" rules for efficiently determining the pre-order as adopted from [27]. We add a new, simple rule to this repertoire that lets us avoid the computationally "expensive"

[4] Using an *instantiation checker* that reuses model-checking results from the last guess.

rules using assumptions from [27]. The information needed to apply this new rule readily comes from parameter lifting as we will see.

*Ordering states for local monotonicity.* Let us consider a conceptual example showing how a pre-order on states can be used for determining local monotonicity.

*Example 6.* Consider the pMC $\mathcal{M}_2$ in Fig. 2(c). We reason backwards that both states are locally monotone increasing in $p$. First, observe that $\ddot\smile$ has a higher probability to reach the target (1) than $\ddot\frown$ (0). Now, in $s_1$, increasing $p$ will move more probability mass to $\ddot\smile$, and hence, it is locally monotone. Furthermore, we know that the probability from $s_1$ is between $\ddot\smile$ and $\ddot\frown$. Now, for $s_0$ we can use that increasing $p$ moves more probability mass to $s_1$, which we know has a higher probability to reach the target than $\ddot\frown$.

As in [27], we determine local monotonicity by ordering states according to their reachability probability.

**Definition 6 (Reachability order).** *A relation* $\preceq_{R,T} \subseteq S \times S$ *is a* reachability order with respect to $T \subseteq S$ and region $R$ *if for all* $s, t \in S$*:*

$$s \preceq_{R,T} t \quad \textit{implies} \quad \big(\forall \vec{u} \in R.\ \mathsf{Pr}^{s\to T}(\vec{u}) \leq \mathsf{Pr}^{t\to T}(\vec{u})\big).$$

*The order* $\preceq_{R,T}$ *is called* exhaustive *if the reverse implication also holds.*

The relation $\preceq_{R,T}$ is a reflexive (aka: non-strict) pre-order. The exhaustive reachability order is the union of all reachability orders, and always exists. Unless stated differently, let $\preceq$ denote the exhaustive reachability order. If the successor states of a state $s$ are ordered, we can conclude local monotonicity in $s$:

**Lemma 1.** *Let* $s, s_1, s_2 \in S$ *with* $\mathcal{P}(s, s_1) = x$ *and* $\mathcal{P}(s, s_2) = 1-x$*. Then:*

$$\textit{for each region } R: \qquad s_2 \preceq_{R,T} s_1 \quad \textit{implies} \quad \mathsf{Pr}^{s\to T}{\uparrow}_x^{\ell,R}.$$

This result suggests to look for a so-called "sufficient" reachability order:

**Definition 7 (Sufficient reachability order).** *A reachability order* $\preceq$ *is* sufficient *for parameter* $x$ *if for all states* $s$ *with* $\mathsf{occur}(s) = \{x\}$ *and* $s_1, s_2 \in$ $\mathsf{succ}(s)$ *it holds:* $(s_1 \preceq s_2 \vee s_2 \preceq s_1)$*.*

Phrased differently, the reachability order $\preceq$ is sufficient for $x \in V$ if $(\mathsf{succ}(s), \preceq)$ is a total order for all $s$ that have transitions labelled with $x$. Observe that in contrast to an exhaustive order, a sufficient order does not need to exist.

*Ordering states efficiently.* Def. 6 provides a conceptually simple scheme to order states $s_1$ and $s_2$: compute the rational functions $\mathsf{Pr}^{s_1\to T}$ and $\mathsf{Pr}^{s_2\to T}$, and compare them. As the size of these multivariate rational functions can be exponential in the number of parameters [16], this is not practically viable. To avoid this, [27] has identified a set of *rules* that provide sufficient criteria to order states. Some of these rules are conceptually based on the underlying graph of a pMC and are computationally cheap; other rules reason about (a partial representation of) the full rational function $\mathsf{Pr}^{s_1\to T}$ and are computationally expensive.

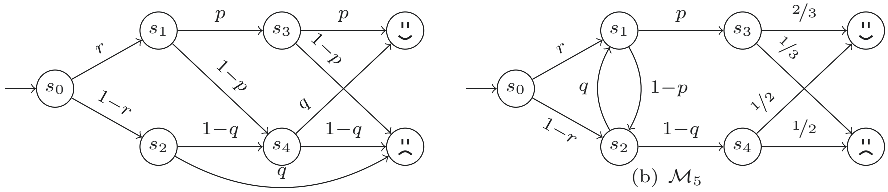

(a) $\mathcal{M}_4$ (b) $\mathcal{M}_5$

**Fig. 5.** Non-trivial pMCs for deducing monotonicity.

*Example 7.* Using bounds avoids expensive rules: See $\mathcal{M}_4$ in Fig. 5(a). Let $R = \{\vec{u}(q) \in [1/2, 3/4], \vec{u}(p) \in [1/2, 2/3]\}$. Using the solution functions $p^2 + (1-p) \cdot q$ and $q \cdot (1-q)$ for $s_1$ and $s_2$ yields $s_2 \preceq s_1$ on $R$. Such a rule is expensive, but the cheaper graph-based rules analogous to Ex. 6 are not applicable. However, when we use bounds from parameter lifting, we obtain $U_R(s_2) = 3/8$ and $L_R(s_1) = 1/2$, we observe $U_R(s_2) \leq L_R(s_1)$ and thus $s_2 \preceq s_1$ on $R$. Bounds also just simplify graph-based reasoning, in particular in the presence of cycles. Consider $\mathcal{M}_5$: As $L_R(s_3) \geq U_R(s_4)$, with reasoning similar to Ex. 6, it follows that $s_2 \preceq s_1$, and we immediately get results about monotonicity.

Our aim is to avoid applying the expensive rules from [27] by imposing a new — and thanks to parameter lifting — cheap rule. To obtain this rule, we assume for state $s$ and region $R$ to have bounds $L_R(s)$ and $U_R(s)$ at our disposal satisfying

$$L_R(s) \;\leq\; \mathsf{Pr}^{s \to T}(\vec{u}) \;\leq\; U_R(s) \quad \text{for all } \vec{u} \in R\ .$$

Such bounds can be trivially assumed to be 0 and 1 respectively, but the idea is to obtain tighter bounds by exploiting the parameter lifter. This will be further detailed in Section 5. A simple observation on these bounds yields a cheap rule (provided these bounds can be easily obtained).

**Lemma 2.** *For $s_1, s_2 \in S$ and region $R$: $L_R(s_1) \geq U_R(s_2)$ implies $s_2 \preceq_{R,T} s_1$.*

In the remainder of this section, we elaborate some technical details.

*Algorithmic reasoning.* The pre-order $\preceq$ is stored by a representation of its Hasse diagram, referred to as RO-graph. Evaluating whether two states are ordered amounts to a graph search in the RO-graph. We start off with the initial order ☹ $\preceq$ ☺. Then we attempt to apply one of the cheap rules to a state $s$. Lemma 2 provides us with more potential to apply a cheap rule. The typical approach is to do this in a reverse topological order over the RO-graph, such that the successors of $s$ are already ordered as much as possible. If the successor states of $s$ are ordered, then $s$ can be added as a vertex and directed edges can be added between $s$ and its successors. Otherwise, state $s$ is added between ☹ and ☺. This often allows for reasoning analogous to the example. To deal with strongly connected components, rules exist [27] that add states to the order even when not all successors are in the graph. If no cheap rule can be applied, more expensive rules using the rational functions from above or SMT-solvers are applied[5].

## 5 Parameter Lifting with Monotonicity Information

Recall that our aim is to compute some $\lambda \geq \max_{\vec{u} \in R} \mathsf{Pr}^{s \to T}_{\mathcal{M}}(\vec{u}) - \varepsilon$ for some fixed region $R$. In order to do so, we compute $\widehat{\lambda} := \max_{\vec{u} \in \mathsf{relax}(R)} \mathsf{Pr}^{s \to T}_{\mathsf{relax}(\mathcal{M})}(\vec{u})$ on the iMC $\mathsf{relax}(\mathcal{M})$ obtained by relaxing the pMC $\mathcal{M}$. We discuss how to speed up this computation using local monotonicity information. In the remainder, let $\mathcal{D}$ denote $\mathsf{relax}(\mathcal{M})$ and $I$ denote $\mathsf{relax}(R)$. As we consider simple iMCs, let state $s$ with $\mathcal{P}(s, s_1) = x_s$ and $\mathcal{P}(s, s_2) = 1 - x_s$ where the parameter $x_s$ does not occur on other transitions. Assume the lower (upper) bound on $x_s$ is $l_s$ ($u_s$).

*Analyzing (simple) iMCs.* An iMC induces a maximal reachability bound by substituting every $x_s$ with either $l_s$ or $u_s$. Formally, let $\mathcal{V}(I)$ denote the corner points of the interval $I$. Then,

$$\max_{\vec{u} \in I} \mathsf{Pr}^{s \to T}_{\mathcal{D}}(\vec{u}) \;=\; \max_{\vec{u} \in \mathcal{V}(I)} \mathsf{Pr}^{s \to T}_{\mathcal{D}}(\vec{u}).$$

Thus, to maximise the probability to reach $T$, in every state $s$ either the lower or the upper bound of parameter $x_s$ has to be chosen. This induces $\mathcal{O}(2^{|S|})$ choices. They can be efficiently navigated by interpreting these choices as nondeterministic choices, interpreting the iMC as a Markov decision process (MDP) [25].

*Local monotonicity helps.* Assume local monotonicity establishes $s_1 \preceq s_2$, i.e., the reachability probability from $s_2$ is at least as high as from $s_1$. To maximise the reachability probability from $s$, the parameter $x_s$ should be minimised. Contrary, if $s_2 \preceq s_1$, parameter $x_s$ should be maximised. Thus, every local monotonicity result halves the amount of vertices that we are maximising over.

*Example 8.* Consider the iMC $\mathcal{M}_3$ in Fig. 3(a), which is the relaxation of the pMC $\mathcal{M}_1$ in Fig. 2(a). There are four combinations of lower and upper bounds that need to be investigated to compute the upper bound. Using local monotonicity, we deduce that $q$ should be as low as possible and $p$ as high as possible. Rather than evaluating a MDP, we thus obtain the same upper bound on the reachability probability in $\mathcal{M}_1$ by evaluating a single parameter-free Markov chain.

*Accelerating value iteration.* Parameter lifting [25] creates a single MDP — a comparatively expensive operation — and instantiates this MDP based on the region $R$ to be checked. For computing the bound $\widehat{\lambda}$, specifically, it uses value iteration. Roughly, this means that for each state we start with either its lower or upper bound. The instantiated MC is then checked. Then, all bounds that can

[5] In an attempt to reduce the cost of these rules, the algorithm allows for deferring proof obligations in the form of assumptions. This is detailed in [27]. For this paper, however, the only relevant aspect is that these rules are computationally expensive.

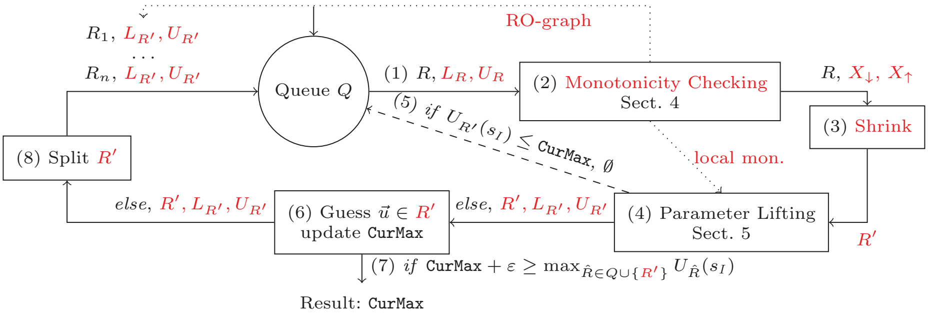

**Fig. 6.** The symbiosis of monotonicity checking and parameter lifting. Red are new elements compared to the vanilla approach in Fig. 4.

be improved by switching from lower to upper bound or vice versa are swapped. This procedure terminates with the optimal assignment to all bounds. We exploit the local monotonicity in this value iteration procedure by fixing the chosen bounds at locally monotonic states.

## 6 Lifting and Monotonocity, Together

In this section, we give a more detailed account of our approach, i.e., we will zoom in into Fig. 1 resulting in Fig. 6. In particular, we detail the divide-and-conquer block. This loop is a refinement (indicated in red in Fig. 6) of Fig. 4. We first give an overview, before discussing some aspects in greater detail.

**Overall algorithm** The approach considers *extended* regions, i.e., a region $R$ is equipped with state bounds $L_R(s)$ and $U_R(s)$ such that $L_R(s) \leq \mathsf{Pr}_{\mathcal{M}}^{s \to T}(\vec{u}) \leq U_R(s)$ for every state $s$, and with monotonicity information about the monotonic increasing (and decreasing) parameters on $R$. Initially the input region $R$ is extended with $L_R(s) = 0, U_R(s) = 1$ for every $s$, and empty monotonicity information. Additionally, we initialize a conservative approximation for the maximum probability CurMax so far as 0. Extended regions are stored in the priority queue $Q$ where $U_R(s_I)$ are used as priority. We discuss details below. Once initialised, we start an iterative process to update the conservative approximation of $L_R$ and $U_R$.

First, (1) a region $R$ and the associated reachability order stored as RO-graph is taken from the queue $Q$ and (2) its monotonicity is computed while using the annotated bounds $L_R$ and $U_R$. Let $X_\uparrow^R$ denote globally monotonic increasing parameters on $R$, and similarly, $X_\downarrow^R$ denote decreasing parameters on $R$. For brevity, we omit the superscript $R$ in the following.

As a next step, we (3) *shrink* a region based on global monotonicity. We define the region $\mathsf{Shrink}_{X_\uparrow, X_\downarrow}(R)$ as follows: $\mathsf{Shrink}_{X_\uparrow, X_\downarrow}(R)(x) = \ell_x$ if $x \in X_\downarrow$,

$\mathsf{Shrink}(R)(x) = u_x$ if $x \in X_{\uparrow}$, and $\mathsf{Shrink}(R)(x) = R(x)$ otherwise. In the remainder of this section, let $R'$ denote $\mathsf{Shrink}_{X_{\uparrow},X_{\downarrow}}(R)$. Observe that we can safely discard instantiations in $R \setminus R'$, as $\max_{\vec{u} \in R} \mathsf{Pr}^{s \to T}_{\mathcal{M}}(\vec{u}) = \max_{\vec{u} \in R'} \mathsf{Pr}^{s \to T}_{\mathcal{M}}(\vec{u})$.

Next, we (4) analyse the region $R'$ to get bounds $L_{R'}, U_{R'}$ using parameter lifting and using the local monotonicity information from the monotonicity check. We make two observations: First, it holds that $L_R(s) \leq L_{R'}(s)$ and $U_{R'}(s) \leq U_R(s)$ for every $s$: Thus, there is no regret in analysing $R'$ rather than $R$. Also, consider that if all parameters are globally monotone, the region $R'$ is a singleton and straightforward to analyse.

If (5) $U_{R'}(s_I) \leq \texttt{CurMax}$, then we discard $R'$ altogether and go to (1). Otherwise, we (6) guess a candidate $\vec{u} \in R'$, and set $\texttt{CurMax}$ to $\max(\texttt{CurMax}, \mathsf{Pr}^{s \to T}_{\mathcal{M}}(\vec{u}))$. If (7) $\texttt{CurMax} + \varepsilon \geq \max_{\hat{R} \in Q \cup \{R'\}} U_{\hat{R}}(s_I)$, then we have solved our problem statement by returning $\texttt{CurMax}$. Otherwise, we cannot yet give a conclusive answer, and need to refine our analysis. To that end, we (8) *split* the region $R'$ into smaller (rectangular) regions $R_1, \ldots, R_n$. Note that these sub-regions first inherit the bounds of the region $R'$; their bounds are refined in a subsequent iteration (if any). Termination in the limit (i.e., convergence of the lower and upper bound to the limit) follows from the termination of monotonicity checking and the termination of the loop in Fig. 4.

**Incrementality** A key aspect in tuning iterative approaches is the concept of incrementality; i.e., reusing previously computed information in later computation steps. Parameter lifting is already incremental [25] by reusing the MDP structure in an efficient manner. Let us address incrementality for the monotonicity checker. Notice that all monotonicity information and all bounds that are computed for region $R$ carry over to any $\hat{R} \subseteq R$. In particular, $s \preceq_{R,T} s'$ implies $s \preceq_{\hat{R},T} s'$ . Furthermore, our monotonicity checker may give up in an iteration if no cheap rules to determine monotonicity can be applied. In that case, we annotate the current reachability order such that after refining bounds, in a subsequent iteration, we can quickly check where we gave up in a last iteration, and whether refined bounds allow progress in constructing the reachability order. Notice that in principle, we have to duplicate the order for each region. However, we do this only until the monotonicity checker does not stabilize. The checker stabilizes, e.g., if an order is sufficient. Once the checker stabilized, we do not duplicate the order anymore (as no more local or global monotonicity can be deduced).

**Heuristics** Our approach allows for several choices in the implementation. Whereas the correctness of the approach does not depend on how to resolve these choices, they have a significant influence on the performance. We discuss (what we believe to be) the most important choices, and how we resolved these choices in the current implementation.

*Initialising* $\texttt{CurMax}$*.* Previously Storm was applicable only to few parameters and generously initialized $\texttt{CurMax}$ by sampling all vertices $\mathcal{V}(R)$, which is exponential in the number of parameters. To scale to more parameters, we discard this sampling. Instead, we sample for each parameter independently to find out which parameters are definitely not monotone. Naturally, we skip parameters already known to be monotone. We select sample points as follows. We distribute the 50 points evenly along the dimension of the parameter. All other parameter values are fixed: Non-monotonic parameters are set to their middle point in their interval (as described by the region). Monotone parameters are set at the upper (lower) bound when possibly monotone increasing (decreasing).

*Updating `CurMax`.* To prove that `CurMax` is close to the maximum, it is essential to find a large value for `CurMax` fast. In our experience, sampling at too many places within regions yields significant overhead, but taking $L(s_I)$ is a too pessimistic way to update `CurMax`. To update `CurMax`, we select a single $\vec{u} \in R'$ in the middle of region $R'$. As we may have shrunk the region $R$, the middle of $R'$ does not need to coincide with the middle of $R$, which yields behavior different from the vanilla refinement loop.

*How and where to split?* There are *two* important splitting decisions to be made. First, we need to select the dimensions (aka: parameters) in which we split. Second, we need to decide where to split along these dimensions. We had little success with trivial attempts to split at better places, so the least informative split in the middle remains our choice for splitting. However, we have changed where (in which parameter or dimensions) to split. Naturally, we do not (need to) split in monotonic parameters. Previously, parameter lifting split in every dimension at once. Let us illustrate that this quickly becomes infeasible: Assume 10 parameters. Splitting the initial region once yields 1024 regions. Splitting half of them again yields $> 500{,}000$ regions. Instead, we use *region estimates*, which are heuristic values for every parameter, based on the implementation of [19]. These estimates, provided by the parameter lifter, essentially consider how well the policy on the MDP (selecting upper or lower bounds in the iMC) agrees with the dependencies induced by a parameter: The more it agrees, the lower the value. The key idea is that one obtains tighter bounds if the policy adheres to the dependencies induced by the parameters[6]. We split in the dimension with the largest estimate. If the region estimate is smaller than $10^{-4}$, then we split in the dimension of $R$ with the widest interval.

*Priorities in the region queue.* Contrary to [25], we want to find the extremal value within the complete region, rather than partitioning the state space. Consequently, the standard technique splits based on the *size* of the region, and de-facto, a breadth-first search. When we split a region, we prioritize the subregions $\hat{R} \subseteq R'$ with $U_{R'}(s_I)$, as $U_{\hat{R}}(s_I) \leq U_{R'}(s_I)$. We use the age of a region to break ties. Here, a wild range of exploration strategies is possible. To avoid overfitting, we refrain in the experiments from weighting different aspects of the region, but the current choice is likely not the final answer.

[6] Technically, the value is computed as the sum of the differences between the local lower and upper bound on the reachability probability over all states with this parameter.

**Table 1.** Overview of the experimental results comparing vanilla parameter lifting to the integrated approach

| | | | | | $\varepsilon$: 0.1 | | | | | $\varepsilon$: 0.05 | | | | |
|---|---|---|---|---|---|---|---|---|---|---|---|---|---|---|
| | | | | | integrated | | | vanilla | | integrated | | | vanilla | |
| **name** | **instance** | **#states** | **#trans** | $\|V\|$ | **# i** | **# i$_b$** | **t** | **#i** | **t** | **# i** | **# i$_b$** | **t** | **#i** | **t** |
| NRP | (5,1) | 56 | 75 | 5 | 469 | 2 | <1 | 2575 | <1 | 5143 | 2 | **<1** | 48701 | 3 |
| | (10,1) | 186 | 250 | 10 | 66219 | 2 | **11** | 512909 | 85 | 7168029 | 2 | **1594** | | TO |
| | (12,1) | 259 | 348 | 12 | 425643 | 2 | **98** | 3304325 | 757 | | | TO | | TO |
| | (13,1) | 300 | 403 | 13 | 1103811 | 2 | **299** | | TO | | | TO | | TO |
| | (14,1) | 344 | 462 | 14 | 2608869 | 2 | **718** | | MO | | | MO | | MO |
| | (15,1) | 391 | 525 | 15 | | | TO | | MO | | | MO | | MO |
| EVADE | (1,2,0,1) | 129 | 249 | 40 | 0 | 2 | **<1** | 2410 | 2 | 0 | 2 | **<1** | 4619 | 4 |
| | (1,2,3,1) | 513 | 993 | 160 | 0 | 2 | **3** | | MO | 0 | 2 | **3** | | MO |
| | (1,2,0,2) | 425 | 842 | 141 | 0 | 2 | **2** | | MO | 0 | 2 | **2** | | MO |
| | (1,2,3,2) | 1697 | 3362 | 561 | 0 | 2 | **21** | | MO | 0 | 2 | **22** | | MO |
| Herman | (11,10) | 21500 | 242926 | 1 | 3 | 3 | 3 | 3 | **2** | 9 | 3 | 14 | 9 | **3** |
| | (11,15) | 31740 | 369706 | 1 | 5 | 3 | 14 | 5 | **3** | 11 | 3 | 25 | 11 | **5** |
| | (13,15) | 126888 | 1713246 | 1 | 7 | 5 | 44 | 7 | **18** | 11 | 6 | 440 | 11 | **24** |
| | (13,25) | 208808 | 2889206 | 1 | 7 | 5 | 91 | 7 | **31** | 11 | 6 | 1415 | 11 | **41** |
| | (13,35) | 290728 | 4065166 | 1 | 5 | 4 | 128 | 5 | **35** | | | TO | 11 | **54** |
| Maze | (25) | 360 | 660 | 24 | 0 | 2 | <1 | 1 | <1 | 0 | 2 | <1 | 40 | <1 |
| | (1000) | 14985 | 26985 | 999 | 0 | 2 | 1 | 1 | **<1** | 0 | 2 | **1** | | MO |
| | (10000) | 149985 | 269985 | 9999 | 0 | 2 | 166 | 1 | **<1** | 0 | 2 | **182** | | TO |

*Obtaining bounds for the monotonicity checker.* While the baseline loop only computes upper-bounds, we use lower bounds to boost the monotonicity checking. We currently run these bounds until the monotonicity checker has stabilized. We observe that, mostly due to numerical computations, the time that the lower bounds take can be significant, but the overhead and the merits of getting larger lower bounds are hard to forecast.

## 7 Empirical Evaluation

*Setup.* We investigate the performance of the extended divide-and-conquer approach presented in Fig. 6. We have implemented the algorithm explained above in the probabilistic model checker Storm [11]. We compare its performance with vanilla parameter lifting, outlined in Fig. 4, as baseline. Both versions use the same underlying data structures and version of Storm. All experiments were executed on a single core Intel Xeon Platinum 8160 CPU. We did neither use any parallel processing nor randomization. We used a time out of 1800s and a memory limit of 32GB. We exclude model-building times from all experiments and emphasize that they coincide for the vanilla and new implementations.

*Benchmarks and results.* The common benchmarks *Crowds*, *BRP*, and *Zeroconf* have only globally monotonic parameters (and only two). Using monotonicity, they become trivial. The structure of *NAND* and *Consensus* makes them not amenable to monotonicity checking, and the performance mostly resembles the baseline. We selected additional benchmarks from [2], [23], and [18], see below. The models from the latter two sources are originally formulated as partially observable MDPs and were translated into pMC using the approach in [19].

Table 1 summarizes the results for benchmarks identified by their name and instance. We list the number of states, transitions and parameters of the pMC.

For each benchmark, we consider two values for $\varepsilon$: $\varepsilon$=0.05 and $\varepsilon$=0.1. For each $\varepsilon$, we consider the time $t$ required and the number ($\mathbf{i}$) of iterations that the integrated loop and the baseline require. For the integrated loop, we additionally provide the number ($\mathbf{i}_b$) of extra (lower bound) parameter lifting invocations needed to assist the monotonicity checker.

*Discussion of the results.* We make the following observations.

- NRP: this model is globally monotonic in all its parameters. Our monotonicity checker can find this one parameter. The integrated approach is an order of magnitude faster on all instances, scaling to more parameters.
- Evade: this model is globally monotonic in some of its parameters. Our monotonicity check can find this monotonicity for a subset. The integrated approach is faster on all instances, as a better initial `CurMax` is guessed based on the results from the monotonicity checker.
- Herman's protocol: this is a less favourable benchmark for the integrated approach as only one parameter is not globally monotonic. The calculation of the bounds for the monotonicity checking yields significant overhead.
- Maze: this model is globally monotonic in all its parameters. This can be found directly by the monotonicity checker, so we are left to check a single valuation. This valuation is also provably the optimal valuation.

In general, for $\varepsilon$=0.1, the number of regions that need to be considered is relatively small and guessing an (almost) optimal value is not that important. This means that the results are less volatile to changes in the heuristic. For $\varepsilon$=0.05, it is significantly trickier to get this right. Monotonicity helps us in guessing a good initial point. Furthermore, it tells us in which parameters we should and should not split. Therefore, we prevent unnecessary splitting in some of the parameters.

## 8 Conclusion and Future Work

This paper has presented a new technique for tackling the optimal synthesis problem: what is the instance of a parametric Markov chain that satisfies a reachability objective in an optimal manner? The key concept is a deep interplay between parameter lifting, the favourable technique so far for this problem, and monotonicity checking. Experiments showed encouraging results: speed ups of up to two orders of magnitude for various benchmarks, and an increased number of parameters. Future work consists including advanced sampling techniques and applying this approach to other application areas such as optimal synthesis and monotonicity in probabilistic graphical models [26] and hyper-properties in security [1].
